# Supplementary material for: Protein catabolites as blood-based biomarkers of aging physiology: Findings from the Dog Aging Project
Source: bioRxiv. 2024 Oct 21:2024.10.17.618956. Preprint. [Version 1] doi: 10.1101/2024.10.17.618956 (PMC11526923; doi:10.1101/2024.10.17.618956)
Supplement: Supplement 2 [file NIHPP2024.10.17.618956v1-supplement-2.pdf]

bioRxiv preprint doi: https://doi.org/10.1101/2024.10.17.618956; this version posted October 21, 2024. The copyright holder for this preprint (which was not certified by peer review) is the author/funder, who has granted bioRxiv a license to display the preprint in perpetuity. It is made available under aCC-BY 4.0 International license.

Figure S1

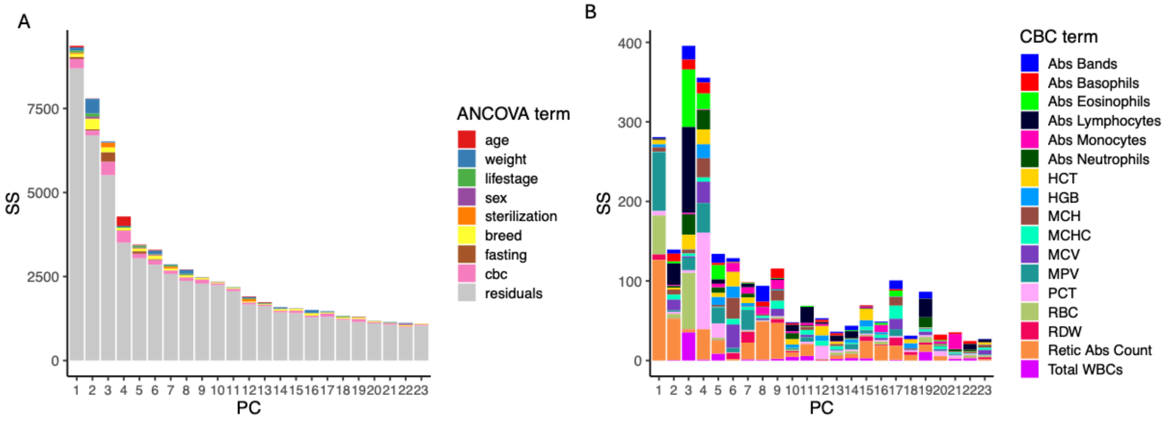

Figure S1 The multivariate dog plasma metabolome *(A) ANOVA sum of squares (SS) among the covariates (term) within each of the first 23 principal components (PC) of the plasma metabolome. The residual SS not accounted for by the terms is shown in gray. In (A) the SS from the 17 CBC traits are combined and indicated by the term 'CBC' (pink). (B) The SS for each of the 17 CBC traits (CBC term) across the first 23 PCs of the metabolome.*

bioRxiv preprint doi: https://doi.org/10.1101/2024.10.17.618956; this version posted October 21, 2024. The copyright holder for this preprint (which was not certified by peer review) is the author/funder, who has granted bioRxiv a license to display the preprint in perpetuity. It is made available under aCC-BY 4.0 International license.

Figure S2

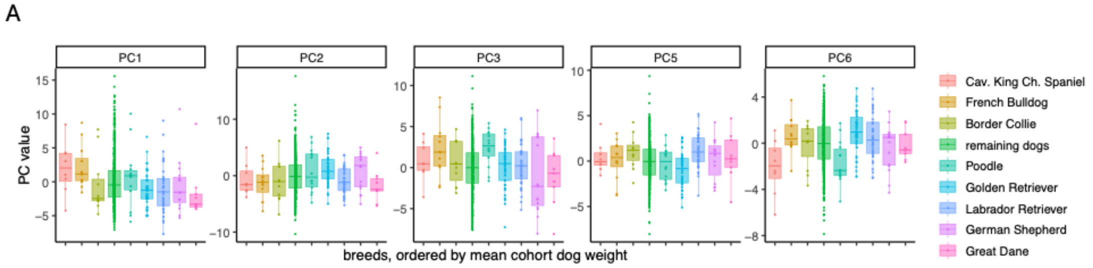

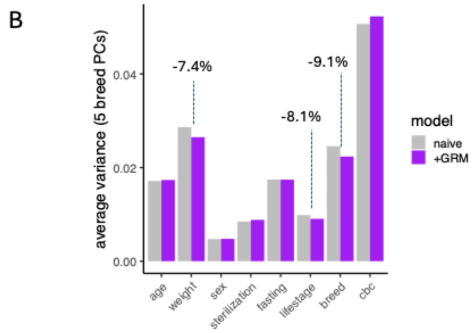

Figure S2 Breed has complex effects on the metabolome, partly accounted for by relatedness *(A) The 5 principal components (PCs) with effects of the 8 common breeds (ANCOVA P<0.05) plotted by breed, including all other remaining dogs. Within each plot, breeds are ordered by the mean weight of the dogs in each breed (n=8 to 44 cohort dogs per breed). (B) The average variance among the 5 PCs in (A) that was accounted for by the fixed effects indicated on the x-axis (BLUEs, Methods) in models that either include a random effect of relatedness by including the gene relatedness matrix (+GMR), or not (naïve, Methods). The percent reduction in average variance is indicated for the three most affected terms.*

bioRxiv preprint doi: https://doi.org/10.1101/2024.10.17.618956; this version posted October 21, 2024. The copyright holder for this preprint (which was not certified by peer review) is the author/funder, who has granted bioRxiv a license to display the preprint in perpetuity. It is made available under aCC-BY 4.0 International license.

Figure S3

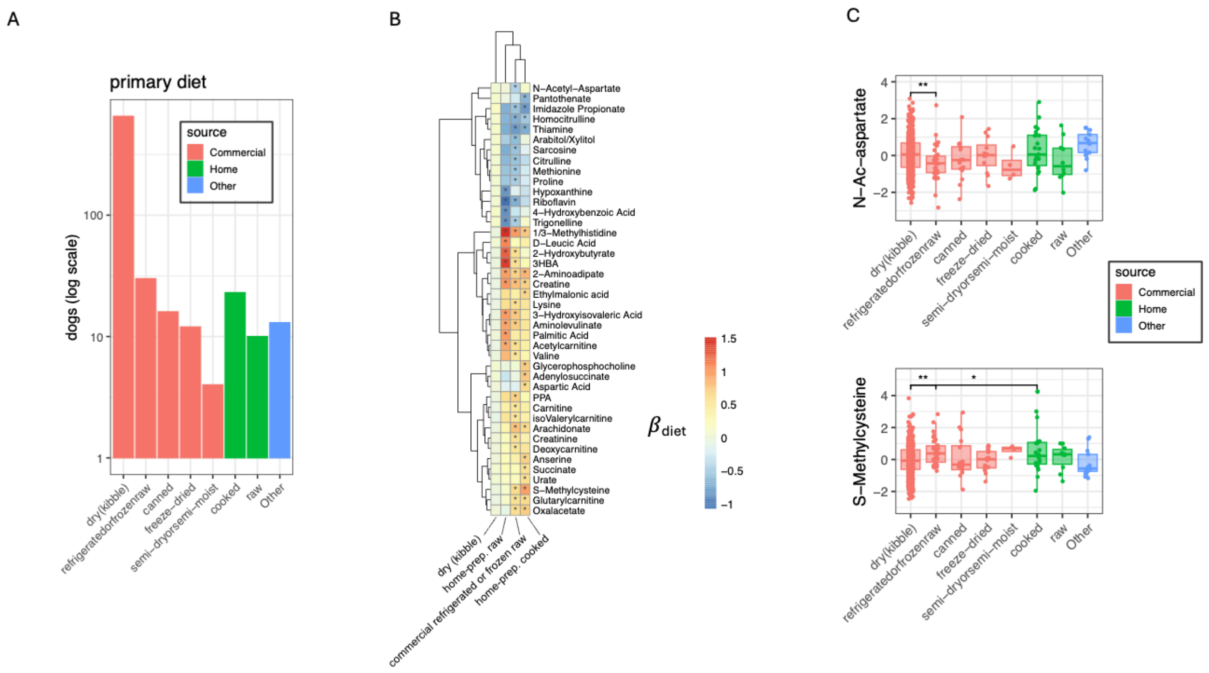

Figure S3 Primary diet composition does not explain variation in post-translationally modified amino acids in plasma. *(A) The distribution of the primary diet type among owner survey responses of 761 dogs, with counts plotted on a log scale. Diet components were divided into those commercially sourced (Commercial), home prepared (Home), or of some other type (Other). Non-responses were omitted, and so Other reflects diet components that do not fit one of the seven categories or where diet was not consistent. (B) A heatmap showing the effect of diet type on each metabolite (β_{diet}) in comparison to dry kibble in a mixed model to control for age, weight, and other covariates (Methods). Alongside the metabolite values among the reference diet, only metabolites and diets that had at least one effect are shown (β_{diet}≠0, FDR < 0.05, asterisks). (C) N-Ac-aspartate and S-methylcysteine were the only post-translationally modified amino acids to associate with any primary diet component (FDR<5%, asterisks).*

bioRxiv preprint doi: https://doi.org/10.1101/2024.10.17.618956; this version posted October 21, 2024. The copyright holder for this preprint (which was not certified by peer review) is the author/funder, who has granted bioRxiv a license to display the preprint in perpetuity. It is made available under aCC-BY 4.0 International license.

Figure S4

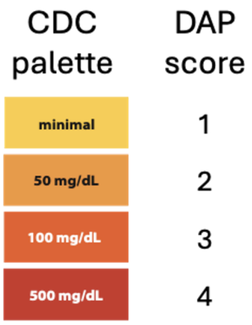

Figure S4 Hemolysis Reference Palette. *The colorimetric reference palette used by the Dog Aging Project (DAP) to assess plasma sample hemolysis. Plasma samples in polypropylene microfuge tubes were compared to the Center for Disease Control and Prevention (CDC) palette and given a score from 1 to 4. Samples with a score of 4 were not analyzed.*

bioRxiv preprint doi: https://doi.org/10.1101/2024.10.17.618956; this version posted October 21, 2024. The copyright holder for this preprint (which was not certified by peer review) is the author/funder, who has granted bioRxiv a license to display the preprint in perpetuity. It is made available under aCC-BY 4.0 International license.

Table S2: Summary of Complete Blood Count, Serum Chemistry and Urinalysis Variables

Analysis	Variable	Units	N	Mean	Sd	Min	Max	Kurtosis	Skewness	variableCode
CBC	Total WBCs	K uL^{-1}	825	7.8	2.3	2.9	21	6.5	1.2	krt_cbc_total_wbcs
CBC	Abs Bands	K uL^{-1}	825	0.0019	0.022	0	0.5	328	17	krt_cbc_abs_bands
CBC	Abs Neutrophils	K uL^{-1}	825	1.8	0.29	0	2.9	4.7	0.051	krt_cbc_abs_neutrophils
CBC	Abs Lymphocytes	K uL^{-1}	825	0.99	0.3	0.086	2.2	3	0.21	krt_cbc_abs_lymphocytes
CBC	Abs Monocytes	K uL^{-1}	825	0.3	0.17	0	0.92	3.8	0.79	krt_cbc_abs_monocytes
CBC	Abs Eosinophils	K uL^{-1}	825	0.37	0.23	0	1.4	4	0.91	krt_cbc_abs_eosinophils
CBC	Abs Basophils	K uL^{-1}	825	0.003	0.02	0	0.3	104	9.1	krt_cbc_abs_basophils
CBC	RBC	M uL^{-1}	825	7.1	0.75	4.7	10	3.4	-0.13	krt_cbc_rbc
CBC	HGB	g dL^{-1}	825	18	1.8	12	23	3.2	-0.27	krt_cbc_hgb
CBC	HCT	%	825	52	5.4	34	72	3.3	-0.21	krt_cbc_hct
CBC	MCV	fL	825	74	3.3	60	88	4.4	0.32	krt_cbc_mcv
CBC	MCH	pg	825	25	1.1	19	29	4.2	-0.14	krt_cbc_mch
CBC	MCHC	g dL^{-1}	825	33	1	29	40	6.8	0.37	krt_cbc_mchc
CBC	RDW	%	825	2.6	0.051	2.4	2.8	5.7	0.98	krt_cbc_rdw
CBC	MPV	fL	825	2.6	0.26	2	3.7	3.9	0.78	krt_cbc_mpv
CBC	PCT	K uL^{-1}	825	0.27	0.086	0.058	0.67	4.4	0.95	krt_cbc_pct
CBC	Retic Abs Count	K uL^{-1}	825	4	0.56	2.2	5.9	2.6	-0.25	krt_cbc_retic_abs
Chem	Total Protein	g dL^{-1}	825	6.1	0.45	4.7	8	3.7	0.2	krt_cp_total_protein_value
Chem	Albumin	g dL^{-1}	825	3.2	0.26	2.4	4.2	3.7	0.15	krt_cp_albumin_value
Chem	Globulins	g dL^{-1}	825	2.9	0.39	1.9	5.1	4.8	0.68	krt_cp_globulins_value
Chem	Albumin : Globulin Ratio	ratio	825	1.1	0.18	0.5	1.9	3.9	0.28	krt_cp_alb_glob_ratio_value
Chem	Calcium	mg dL^{-1}	825	2.4	0.056	2	2.6	15	-2.1	krt_cp_calcium_value
Chem	Phosphorus	mEq L^{-1}	825	1.5	0.18	0.79	3.2	13	0.48	krt_cp_phosphorus_value
Chem	Magnesium	mg dL^{-1}	825	1	0.081	0.83	1.4	3.3	0.5	krt_cp_magnesium_value
Chem	Glucose	mg dL^{-1}	825	4.6	0.18	2.4	5.4	46	-4.5	krt_cp_glucose_value

bioRxiv preprint doi: https://doi.org/10.1101/2024.10.17.618956; this version posted October 21, 2024. The copyright holder for this preprint (which was not certified by peer review) is the author/funder, who has granted bioRxiv a license to display the preprint in perpetuity. It is made available under aCC-BY 4.0 International license.

Chem	BUN	mg dL^{-1}	825	2.9	0.27	2.1	4.2	5.5	0.62	krt_cp_bun_value
Chem	Creatinine	mg dL^{-1}	825	0.7	0.12	0.33	1.5	7.6	0.8	krt_cp_creatinine_value
Chem	Bilirubin - Total	mg dL^{-1}	825	0.2	0.06	0.095	0.79	17	2.2	krt_cp_bilirubin_total_value
Chem	ALKP	U L^{-1}	825	3.6	0.86	1.8	7.2	5.3	1.3	krt_cp_alkp_value
Chem	ALT	U L^{-1}	825	3.7	0.51	1.6	6	5.4	1	krt_cp_alt_value
Chem	GGT	U L^{-1}	825	1.5	0.24	1.1	4.3	33	4.2	krt_cp_ggt_value
Chem	Amylase	U L^{-1}	825	6.2	0.33	4.9	7.6	3.8	0.34	krt_cp_amylase_value
Chem	Triglycerides	mg dL^{-1}	825	4.2	0.62	3	8.6	11	2.3	krt_cp_triglycerides_value
Chem	Cholesterol	mg dL^{-1}	825	5.5	0.28	4.6	7.2	5.7	0.61	krt_cp_cholesterol_value
Chem	Sodium	mEq L^{-1}	825	147	1.8	137	155	4.9	0.033	krt_cp_sodium_value
Chem	Potassium	mEq L^{-1}	825	4.5	0.36	3.1	6.2	4.3	0.57	krt_cp_potassium_value
Chem	Chloride	mEq L^{-1}	825	4.7	0.023	4.6	4.9	6.2	-0.41	krt_cp_chloride_value
Chem	Sodium : Potassium Ratio	ratio	825	33	2.7	23	48	4.3	0.22	krt_cp_sp_ratio_value
Urinalysis	Specific Gravity	NA	738	1	0.014	1	1.1	2.5	-0.53	krt_urine_sg
Urinalysis	Bilirubin	mg dL^{-1}	738	1.4	0.54	1	3	2.3	0.65	krt_urine_bilirubin
Urinalysis	Blood/HGB	Hb dL^{-1}	738	1.2	0.75	1	5	19	4.1	krt_urine_blood_hbg
Urinalysis	pH	pH	738	6.8	0.74	6	8.5	2.5	0.69	krt_urine_ph
Urinalysis	Protein	mg dL^{-1}	738	1.9	1	1	5	3.6	1	krt_urine_protien
Urinalysis	WBC/HPF	hpf^{-1}	738	2.8	1.5	1	8	4.7	1	krt_urine_wbc_hpf
Urinalysis	RBC/HPF	hpf^{-1}	738	2.2	1.6	1	8	6.1	1.7	krt_urine_rbc_hpf
Urinalysis	Squamous/HPF	hpf^{-1}	738	2.5	1.2	1	8	3.3	0.23	krt_urine_squamous_hpf
Urinalysis	Urothelial/HPF	hpf^{-1}	738	1.5	0.96	1	8	7.8	2	krt_urine_urothelial_hpf
Urinalysis	Fat/HPF	hpf^{-1}	738	3.9	1.2	1	5	2.7	-0.82	krt_urine_fat_hpf

Table S2 Summary of Complete Blood Count, Serum Chemistry and Urinalysis Variables. The names, units and summary statistics: N=number of dogs with available data, SD=standard deviation, and measures of skewness and kurtosis, for variables selected for analysis based on completeness and other factors (Methods). The variable names in the meta data are given (variableCodes). There were 17 variables selected from the complete blood count (CBC) analysis, (variableCodes contain 'krt_cbc'), 21 serum chemistry variables (Chem, variableCodes contain 'krt_cp'), and 10 variables from the Urinalysis (variableCodes contain 'krt_urine').

bioRxiv preprint doi: https://doi.org/10.1101/2024.10.17.618956; this version posted October 21, 2024. The copyright holder for this preprint (which was not certified by peer review) is the author/funder, who has granted bioRxiv a license to display the preprint in perpetuity. It is made available under aCC-BY 4.0 International license.

Figure S5

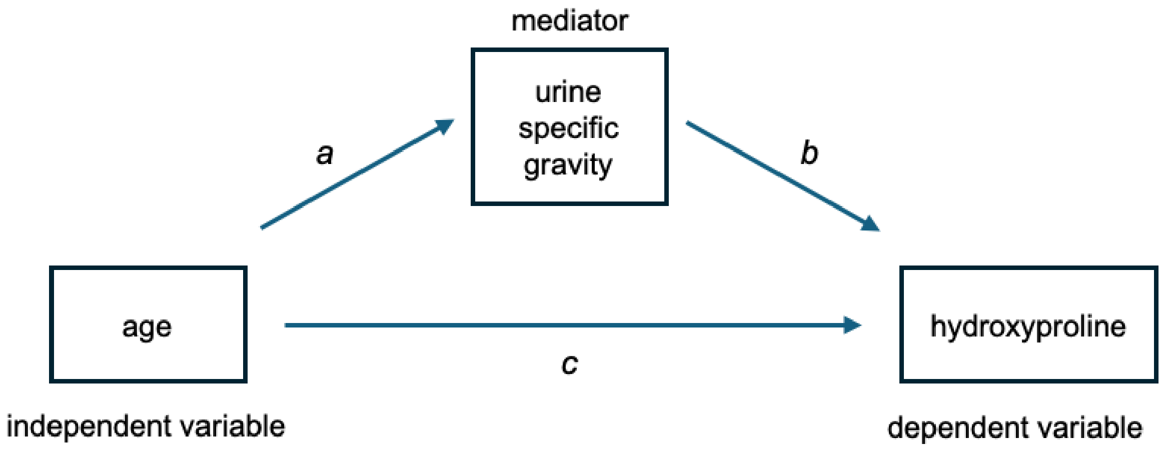

Figure S5 Path representation of causal mediation models *A path representation of a causal mediation model using, as an example, the hypothetical effect of age on hydroxyproline, with urine specific gravity as a potential mediator. The mediation model (path a-b), was compared to a model without urine specific gravity (path c). The total effect was path c, without considering path a-b. To detect causal mediation, after satisfying the assumption that path a was significant, the direct effect (c|b) of urine specific gravity was the effect of path c given path b. The mediation effect was the total effect minus the direct effect, and its significance (γ>0) was tested by bootstrap resampling age in the model (Methods). The proportion mediated was γ divided by the total effect.*
